# Immunoglobulin Gamma-Like Therapeutic Bispecific Antibody Formats for Tumor Therapy

**DOI:** 10.1155/2019/4516041

**Published:** 2019-02-11

**Authors:** Shixue Chen, Lingling Li, Fan Zhang, Yu Wang, Yi Hu, Lei Zhao

**Affiliations:** ^1^National Clinical Research Center for Normal Aging and Geriatric & Department of Oncology & Institute of Geriatric & The Key Lab of Normal Aging and Geriatric, The Second Medical Centre, PLA General Hospital, Beijing, China; ^2^Medical of School & Graduate School, Nankai University, Tianjin, China

## Abstract

Bispecific antibodies (BsAbs) are a sort of dual functional proteins with specific binding to two distinct targets, which have become a focus of interest in antibody engineering and drug development research and have a promising future for wide applications in cancer immunotherapy and autoimmune disease. The key of clinical application and commercial-scale manufacturing of BsAbs is the amenability to assembly and purification of desired heterodimers. Advances in genetic engineering technology had resulted in the development of diverse BsAbs. Multiple recombinant strategies have been used to solve the mispairing problem between light and heavy chains, as well as to enforce accurate dimerization of heterologous heavy chains. There are 23 platforms available to generate 62 BsAbs which can be further divided into IgG-like ones and fragment-based ones, and more than 50 molecules are undergoing clinical trials currently. BsAbs with IgG-like architecture exhibit superior advantages in structure (similar to natural antibodies), pharmacokinetics, half-life, FcR-mediated function, and biological activity. This review considers various IgG-like BsAb generation approaches, summarizes the clinical applications of promising new BsAbs, and describes the mechanism of BsAbs in tumor therapy.

## 1. Introduction

In the 2017 World Health Statistics Report released by the WHO, cancer ranks the second most common cause of death following cardiovascular diseases around the world. One out of every ten deaths is caused by cancer and there is an apparent rising trend in the world [1]. Tumor-specific monoclonal antibodies (mAbs) have revolutionized the treatment of cancer. The combination of tumor-specific mAbs with traditional chemotherapy has greatly extended the patients' survival time and 5-year survival rate. However, the complexity and heterogeneity of cancer limit the further application of tumor-specific mAbs. Most of patients treated with tumor-specific target therapy would no longer benefit with retreatment, and acquired resistance is one of the prime obstacles for the successful treatment of cancer. Thus, there is an urgent need to develop novel antitumor reagents with significant improvement of antitumor efficacy.

Bispecific antibodies (BsAbs) could simultaneously target two different ligands or receptors of vital signaling pathways, which would further improve the selectivity and functionality of antibody, and subsequently enhance the safety and antitumor efficacy [2]. Growing evidences have proved that BsAbs could be a promising reagent against tumor, genetic diseases, and infectious diseases in the near future [3, 4]. Nowadays, two antitumor BsAbs have been approved for clinical use. The first therapeutic BsAb catumaxomab was approved by the European Medicines Agency (EMA) for the treatment of malignant ascites in 2009 [5]. The second BsAb blinatumomab has been approved for adult patients with relapsed or refractory B cell precursor acute lymphoblastic leukemia (ALL) by the United States Food and Drug Administration (FDA) in 2014 [6]. Furthermore, there are more than 110 BsAbs in the course of development and more than 50 BsAbs have been evaluated in clinical trials [7, 8].

As we know, the classical IgG architecture as it was selected during evolution has many advantages for therapeutic application [9]. Natural immunoglobulin gamma (IgG) antibodies consist of two heavy chains with 4 domains (HC, comprising the CH3, CH2, CH1, and VH domains) and two light chains with 2 domains (LC, comprising the CL and VL domains). In natural condition, an antibody with IgG architecture has the capacity to recognize one specific binding site on the target. The BsAbs do not exist in nature and can only be artificially generated. The correct assembly between heterologous HC-HC and LC-LC from different antibodies is critical for the development of BsAbs with the potential for clinic use. As early as the 1990s, the first BsAb was developed for the treatment of ovarian tumors, but due to the failure of phase III clinical trial and the limitation of production technology, the development of BsAb was restricted for a long time [8]. Emerging advances in antibody engineering, which is represented by genetic engineering, have retriggered the craze of BsAb research.

With the development of genetic engineering, up to 23 available platforms have been currently established to generate BsAbs. By using these platforms, there are approximately 60 bispecific molecules developed for various diseases, including cancer and infection diseases. According to the structure of BsAbs [2, 10], it can be divided into two categories: bispecific molecules without Fc segments and bispecific molecules with IgG-like architecture. To our knowledge, the classical IgG architecture, as it was selected during evolution, has many advantages for the therapeutic application of bispecific antibodies [11, 12]. The Fc part is identical to that of a conventional IgG antibody, resulting in IgG-like pharmacokinetic properties and retained effector functions such as the mediation of ADCC through Fc*γ*RIIIa binding. IgG-like size and molecular weight are expected to result in IgG-like diffusion, tumor penetration, and accumulation in comparison with bispecific tetravalent antibodies of higher molecular weight. Concerning these benefits, we will mainly discuss the development of IgG-like BsAbs in this review.

BsAbs with the advantages of dual functions of two different antibodies contain two different antigen-binding sites, which could block or activate two different signaling pathways by dual targeting, or build up a bridge between target cells and functional molecules (cells) for stimulating a directed immune response. The superior efficacy of BsAbs has been clinically validated; numerous pharmaceutical companies (including Amgen, Roche, Pfizer, Chugai, and Genentech) are now focusing on the development of BsAb technologies and therapeutic reagents. According to an estimation, the market of therapeutic BsAbs will grow up to $5.8 billion per year by 2024 [13].

## 2. Various Immunoglobulin Gamma-Like Bispecific Antibody Formats

BsAbs of the IgG-like structure are usually expressed in single cells. The light and heavy chains are theoretically present in systems that are coexpressed in a single cell line. The problem of mismatching is that there may be nine random nonfunctional combinations of HHLLs and one proper assembly of BsAb. However, it is difficult to purify the desired BsAb from the mixture with nine nonfunctional combinations. IGg-like BsAbs containing Fc region can be further divided into asymmetric or symmetric antibodies depending on the structure. Most IGg-like BsAbs are asymmetric, including knobs-into-holes (KiH), CrossMAb, Triomab quadroma, FcΔAdp, asymmetric reengineering technology-immunoglobulin (ART-Ig), BiMAb, Biclonics, Bispecific Engagement by Antibodies based on the T cell receptor (BEAT), DuoBody, Azymetric, XmAb, T cell bispecific antibodies (2 : 1 TCBs), and 1Fab-IgG TDB. On the other hand, IgG-like symmetric BsAbs contain dual variable domain-immunoglobulin (DVD-Ig), FynomAb, and two-in-one/dual action Fab (DAF).

### 2.1. Immunoglobulin Gamma-Like Asymmetric Bispecific Antibodies

#### 2.1.1. Knobs-into-Holes (KiH)

**Figure 1:**
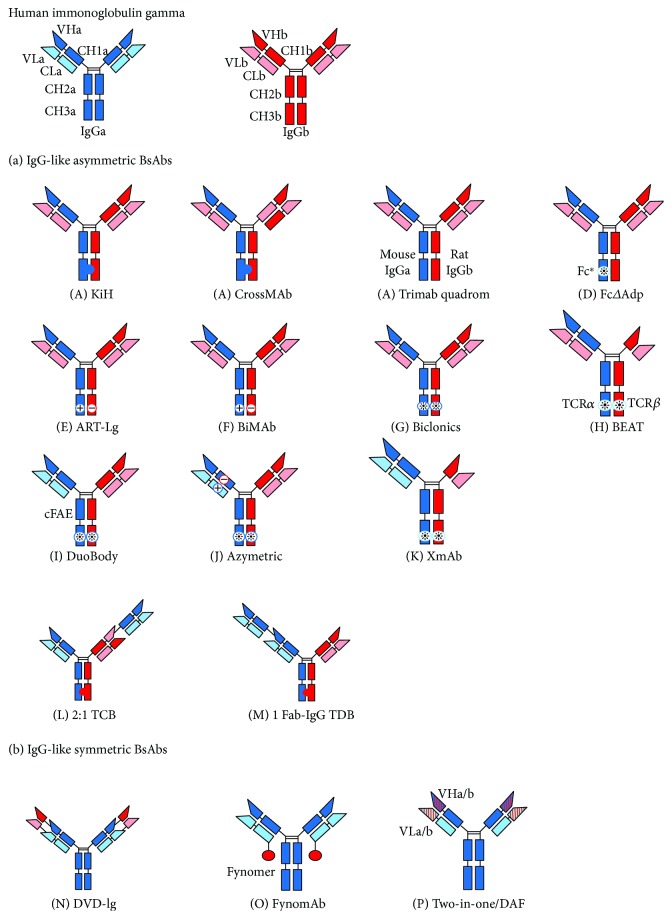
The upper line depicts human immunoglobulin gamma (IgG) parental antibodies IgGa and IgGb. (a) IgG-like asymmetric BsAb platforms including the following: (A) KiH, (B) CrossMAb, (C) Triomab quadroma, (D) FcΔAdp, (E) ART-Ig, (F) BiMAb, (G) Biclonics, (H) BEAT, (I) DuoBody, (J) Azymetric, (K) XmAb, (L) 2 : 1 TCBs, and (M) 1Fab-IgG TDB; (b) IgG-like symmetric BsAb platforms including the following: (N) DVD-Ig, (O) FynomAb, and (P) two-in-one/DAF.

Knobs-into-holes (KiH) technology published in 1996 by Genentech was the first patent approved to facilitate heterologous HCs of BsAb heterodimerization [14] (Figure 1(a), A and Table 1). It was an effective design strategy in avoiding HC mispairing which was one of the key problems in constructing IgG-like BsAbs. By modifying the amino acids of two HCs separately, Ridgway and coworkers generated a matching knob-into-hole structure to promote heterodimerization. A larger amino acid tyrosine was introduced to take the place of a small one threonine in the CH3 domain of one side of the HCs, forming the “knob” (T366Y). Opposite operation was manipulated on the corresponding CH3 area of the other side of the HCs, substitution of a smaller amino acid to generate the “hole” (Y407T). The steric hindrance effect of this modified structure promoted the correct assembly between HCs from different mAbs. Compared with wild type, the correct assembly rate of BsAbs after modification was increased from 57% to 92%, which can meet the requirement of large-scale production. However, structure stability of antibody was reduced as a consequence of modification [14, 15]. In order to overcome this shortcoming, researchers performed random mutation screening by phage display technology to construct a more stable “4 + 2” mode KiH (CW-CSAV) structure: S354C and T366W mutation formed the “knob,” in association with four amino acid mutations forming the “hole” (Y349C, T366S, L368A, and Y407V) and disulfide bond between HC-HC. Although KiH technology can promote heterologous HCs to correctly assemble, it could not avoid the mismatch of LC-HC. The following introduced technology CrossMAb enhances the correct assembling rate of HC-LC [16]. However, KiH technology introduces several hydrophobic amino acids into the interface of CH3-CH3, which could result in nonspecific aggregation and limit the correct assembling rate of CH3-CH3 heterodimer during BsAb generation. Recently, we have successfully developed the “lock-and-key” technology by using computational method to improve the efficiency and correct assembling rate of CH3-CH3 heterodimer. By using structure-based rational design and molecular dynamic simulation, we have redesigned the interface of CH3-CH3 heterodimer by introducing nine hydrophilic polar amino acids and validated the correct assembling rate. Introduction of four amino acid mutations in one side of the CH3 interface forming the “key” (D356K, Q347K, D399K, and K392C) and five amino acid mutations in the other side of the CH3 interface forming the “lock” (K439D/E, K360E, K409D, K392D, and D399C) have exhibited superior correct assembling efficacy than KiH (PCT/CN2017/093787).

#### 2.1.2. CrossMAb

CrossMAb technology has been developed by Roche in 2007, which exchanges LC and HC domains within the Fab of one-half of the BsAb to solve the LC/HC mispairing problem (Figure 1(a), B). The representative products of CrossMAb technology are RG7221 and RG7716, both of which are anti-angiopoietin-2 (Ang-2)/vascular endothelial growth factor (VEGF) BsAbs [17]. There exist two exchanging forms of CrossMAb: the exchange of variable (CrossMAb^VH-VL^) or constant domain (CrossMAb^CH1-CL^) of the Fab between LC/HC.. The CrossMAb technology enables BsAbs of bivalent, trivalent, tetravalent, and also IgG fusion proteins. CrossMAb combined with KiH technology is becoming a versatile platform to product IgG-like BsAbs, and 6 products have already been undergoing clinical studies (RG-6026 [18], RG-7386 [19], RG-7802 [20], RG-7828 [21] in phase I, and RG-7221 [17] and RG-7716 [22] in phase II.  Table 1).

#### 2.1.3. Triomab Quadroma

To solve the mispairing of HC/HC and LC/HC during the development of IgG-like BsAbs, the fusion of two different hybridoma cells harboring different specificities results in a “quadroma” cell line. The “quadroma” cell line has the potential to produce 16 different combinations, including one bispecific molecule with correct assembling and 15 of nonfunctional or monospecific molecules. The triomab quadroma technology developed by Lindhofer and colleagues in 1994 solved the mispairing of LC/HC and HC/HC through the fusion of mouse IgG2a and rat IgG2b hybridomas (Figure 1(a), C and Table 1) [23]. Based on the different binding affinity of mouse and rat Fc part of IgG to protein A, rat/mouse BsAbs can be easily discriminated from the parental mouse and rat antibody and mispairing combination through the purification by protein A [23–25]. In 2017, catumaxomab was voluntarily withdrawn from the European Union (EU) market for commercial reasons (EMA/428877/2017).

#### 2.1.4. FcΔAdp

To solve the LC/HC mispairing problem, FcΔAdp technique using a single common LC and two distinct HCs to form the heterodimeric BsAb was developed by Regeneron in 2009 (Figure 1(a), D). Due to the same light chains, nonfunctional BsAbs resulting from the binding of heavy chains to non-corresponding light chains in the coexpression can be prevented. There are totally three products, two of which are homodimeric for the HCs and one that is the desired heterodimeric BsAb. To collect the desired heterodimeric BsAb, Fc part of antibody with different binding affinity for protein A was employed. By using this technology, REGN-1979, targeting CD3 and CD20 for T cell recruitment, is now undergoing clinical trials in phase I in patients with non-Hodgkin's lymphoma, acute lymphoblastic leukemia, and chronic lymphocytic leukemia (Table 1).

#### 2.1.5. Asymmetric Reengineering Technology-Immunoglobulin (ART-Ig)

Asymmetric reengineering technology-immunoglobulin (ART-Ig) technology was first reported by Chugai in 2005, which overcomes HC/HC mispairing problems through the introduction of electrostatic steering mutations in the CH3 domain interface and achieves correct assembly of LC/HC by utilization of common light chain (Figure 1(a), E). By introducing electrostatic steering mutations into the CH3 of Fc, the heterologous heavy chains from different parental antibodies have strong and more specific interactions between each other, while the homologous heavy chains are hard to form homodimers due to repulsive charge achieved by electrostatic steering mutations [26, 27]. The electrostatic steering mutations facilitate the formation of heterodimers and inhibit the generation of undesired homodimers [28]. Emicizumab was first developed by using this technology, which restores the function of missing activated FVIII by bridging activated FIX and FX to facilitate effective haemostasis in patients with hemophilia A [29]. It was approved by FDA in 2017 for use as routine prophylaxis to prevent or reduce the frequency of bleeding episodes in adults and paediatric patients with hemophilia A (congenital FVIII deficiency) with FVIII inhibitors. Another product, ERY-974, targeting cluster of differentiation protein 3 (CD3) and Glypican 3 (GPC3) for the treatment of solid tumors, is currently undergoing clinical trials in phase I [30].

#### 2.1.6. BiMAb

By using the similar method of ART-Ig, BiMAb reported by OncoMed in 2009 utilizes different electrostatic steering mutations in the CH3 of Fc part to solve the HC/HC mispairing problem. A single common light chain was used in this technology to prevent the mispairing of LC/HC (Figure 1(a), F and Table 1). OMP-305B83 generated by this platform is a BsAb targeting Notch pathway ligand delta-like ligand 4 (DLL4) and VEGF, which is undergoing a phase 1a clinical study for patients with previously treated solid tumors (including ovarian cancer, endometrial cancer, breast cancer, and pancreatic cancer) (Table 1). Preclinical data have showed that OMP-305B83 exhibited excellent tumor killing biological activity in human xenograft models [31].

#### 2.1.7. Biclonics

To generate bispecific antibody with a single human common light chain, a transgenic mouse was developed by Merus in 2012, termed MeMo [32], which took advantage of electrostatic steering effects to promote the heterdimerization of human HCs and used a single human common light chain to avoid HC/LC mispairing in the process of engineering fully integrated IgG-like BsAbs [33, 34] (Figure 1(a), G and Table 1). There are three candidate drugs generated by Biclonics currently undergoing clinical studies. MCLA-117 [35], targeting C-type lectin domain family 12 member A (CLEC12A) and CD3, has demonstrated promising effects in the treatment of acute myeloid leukemia in phase I (Table 1). MCLA-128 [36], targeting human epidermal growth factor receptor-2 (HER-2)/human epidermal growth factor receptor-3 (HER-3), and MCLA-158 [37], targeting leucine-rich repeat-containing G-protein coupled receptor 5 (Lgr5)/EGFR, are currently in clinical phase I/II trials for patients with solid tumors (Table 1).

#### 2.1.8. Bispecific Engagement by Antibodies Based on the T Cell Receptor (BEAT)

The HC/HC mispairing problem can also be solved by BEAT platform, which grafts the TCR constant domain alpha/beta interface onto the CH3 interface [38, 39] (Figure 1(a), H). The BEAT bispecific molecule consists of three parts: a heavy chain, a light chain, and a scFv-Fc. The CH3 domain of a heavy chain consists residues from TCR*α* interface, and the another CH3 domain consists residues from TCR*β* interface. Hence, the heavy chain and Fc-scFv of BEAT BsAb can form specific association avoiding the generation of unwanted HC/HC homodimers. In terms of function, BEAT BsAbs have two distinct antigen-binding sites due to a Fab arm on one side and a scFv on the other side. They also have the biological activities of Fc-mediated functions like ADCC and CDC due to an intact Fc region. The patent application for Glenmark's BEAT platform was filed in 2011 and was published in 2012 (Table 1). GBR-1302 is a kind of BEAT BsAbs, targeting HER2 and CD3 for the treatment of HER2-positive cancers in clinical phase I (Table 1), which has the function of recruiting cytotoxic T lymphocytes (CTLs) to HER2 expressing tumor cells and activates CTLs to kill tumor cells at a very low concentration [40].

#### 2.1.9. DuoBody

Based on the natural process of the Fab arm exchange of human IgG4 isotype in human serum, DuoBody was developed by Genmab in 2010 to overtake the mispairing of HC/HC heterodimer of BsAbs. A single matched point mutation at the interface of CH3-CH3 was introduced to prevent the HC/HC mispairing. In the method, two IgG1 mAbs containing the single matched point mutation are first expression separately. The parental Abs are then mixed and subjected to controlled reducing conditions in vitro that separate the Abs into half-molecules and allow reassembly and reoxidation to form pure IgG1 BsAbs. This technology for generating BsAbs is highly efficient (≥95%) in association with a high stability (especially thermal stability), and the final products have a very low proportion of homodimers (<5%) and multimers (<1%) [41, 42]. Genmab and Janssen collaborate on the DuoBody platform to develop three BsAbs, JNJ-61186372 [43], JNJ-63709178 [44], and JNJ-61178104 [45], which are under evaluation in clinical trial phase I (Figure 1(a), I and Table 1).

#### 2.1.10. Azymetric

By using structure-based rational design and molecular dynamic simulation, Zymeworks has developed Azymetric platform to solve the HC/HC mispairing problem in 2010. T350V, L351Y, F405A, and Y407V were introduced in one side of the CH3 interface, and T350V, T366L, K392L, and T394W were introduced in another side of the CH3 interface. The purity of BsAbs by using this method could be more than 95% (Figure 1(a), J). An orthoFab-Ig BsAb, ZW-25, targeting two nonoverlapping epitopes of HER2, was generated by using Azymetric and orthoFab-Ig methods [46], which is currently in phase I study for patients with HER2-expressing cancers (Table 1) [47].

#### 2.1.11. XmAb

Xencor invented XmAb technology in 2009 to achieve HC/HC heterodimer by introducing four mutations (S364H and F405A in one CH3 domain; Y349T and T394F in another CH3 domain) at the CH3-CH3 interface [48] (Figure 1(a), K and Table 1). By using this technology, XmAb-14045 [49, 50], a Fab-scFv-Fc molecule cotargeting CD3 and CD123, and XmAb13676 [51], cotargeting CD3 and CD20, have been developed, which are currently in phase I clinical trial for the treatment of hematological malignancies and non-Hodgkin lymphoma, respectively (Table 1).

#### 2.1.12. 2 : 1 T Cell Bispecific Antibody (2 : 1 TCB and 1Fab-IgG TDB)

A more recent promising therapeutic approach involves redirecting T cells to attack tumor cells by using BsAbs that bind to a tumor expressing target and common surface component of the T cell receptor (TCR) (e.g., CD3e). Although blinatumomab, a T cell bispecific (TCB) antibody targeting CD19 and CD3e, is approved in relapsed/refractory B cell acute lymphoblastic leukemia (B-ALL) [6] and in clinical trials for non-Hodgkin lymphoma (NHL), it must be administered by continuous infusion due to its short half-life and infusion-related reactions and CNS toxicity is still an issue for blinatumomab in diffuse large B cell lymphoma (DLBCL) [52]. Very recently, Bacac and her colleagues have demonstrated that 2 : 1 TCB (CD20-TCB) with two anti-CD20 Fabs and one anti-CD3 epsilon subunit (CD3e) Fab, in which one of the CD20 Fabs fused directly in a “head-to-tail” fashion to the anti-CD3e Fab via a flexible linker, exhibited superior potency compared with other TCB antibodies based on the classical 1 : 1 IgG format against NHL (Figure 1(a), L) [18]. The 2 : 1 TCB are currently being evaluated in phase I, multicenter study in patients with relapsed/refractory NHL (NCT03075696). In line with these findings, the BCMA-T cell bispecific antibody EM801 with 2 : 1 TCB format showed potent antitumor efficacy against multiple myeloma in the preclinic study [53].

Although early clinical results using T cell-retargeting approaches for treatment of hematological malignancies have generated broad excitement, redirecting T cell activity to eradicate solid tumors is substantially more challenging. The primary barrier to successful treatment of solid tumors with T cell-retargeting therapeutics is the lack of tumor-restricted antigens, which would result in on-target off-tumor adverse effects caused by T cell reactivity to normal tissues expressing the antigen. Recently, Slaga and his colleagues have developed a modified 2 : 1 TCB (1Fab-IgG) with improvement of selectivity and potency against HER2-amplified tumor cells, while sparing cells that express low amounts of HER2 similar to normal human tissues (Figure 1(a), M) [54].

### 2.2. Immunoglobulin Gamma-Like Symmetric Bispecific Antibodies

#### 2.2.1. Dual Variable Domain-Immunoglobulin (DVD-Ig)

Besides asymmetric BsAbs, homodimerized BsAbs could overcome the mispairing problems of HC/HC and LC/HC, which have been getting increasing attention and forming growing numbers of patent applications. The dual variable domain-Ig (DVD-Ig) has been developed by Abbott in 2006, in which the VL and VH domains of an IgG could connect with the similar domains of a second antibody through short peptide linkers [55–57] (Figure 1(b), N). Since the same variable regions of an antibody are added to both N-terminus of IgG antibody, BsAbs produced by the DVD-Ig technology are symmetric and tetravalent, which means a BsAb is bivalent with regard to each antigen. DVD-Ig BsAbs possess the ability to bind four antigens simultaneously, which has a significant meaning in binding cytokines or other proteins with low concentrations and has a better efficacy than suppressing a single target [58]. In addition, DVD-Ig molecules can be generated in traditional mammalian cell expression systems, which means easier to produce and purify as a single molecule and retains the affinity and potency of both parental antibodies.

Representative products of such BsAbs are ABT-122 [59] and ABT-981 [60] both developed by AbbVie (Table 1). ABT122 inactivates the activity of the tumor necrosis factor (TNF) as well as interleukin 17 (IL-17), while ABT-981 binds to the receptor ligands IL-1*α* and IL-1*β*. All these factors play an important role in inflammatory diseases. ABT-122 and ABT-981 are currently undergoing clinical trials in phase II in rheumatoid arthritis and osteoarthritis.

#### 2.2.2. FynomAb

Scaffold proteins have been discovered to exert a critical role in the spatial and temporal assembly of cellular ingredients in the course of biological signaling [61, 62]. Fynomers, a kind of scaffold proteins, are small binding proteins (7 kDa) from the SH3 domain of Fyn kinase. Researchers modified them to obtain binding domains with high affinity to target proteins of interest [63]. In 2014, Covagen publicated that they found another method termed FynomAb for generating IgG-like BsAbs by fusing fynomers to the heavy or light chains of an IgG antibody (Figure 1(b), O and Table 1). Covagen produced COVA-322 on the FynomAb platform via the fusion of IL-17A-binding fynomers to the C-terminus of anti-TNF-*α* molecule adalimumab's light chains (Table 1) [64, 65]. A phase I/II clinical trial of COVA-322 is currently undergoing for the treatment of moderate-to-severe plaque psoriasis. In order to evaluate the toxicity, safety, side-effects, and biological activity of COVA-322, a randomized trial is designed to be ascending single dose, placebo controlled, and double blind [66].

#### 2.2.3. Two-in-One/Dual Action Fab (DAF)

BsAbs generated by the two-in-one/dual action Fab (DAF) technology differ from appending BsAbs constructed by the DVD-Ig or FynomAb that the former achieves bispecificity via some mutations in the variant regions of regular IgG antibodies without any appendage (Figure 1(b), P). The amino acid composition and order of three regions of each VH and VL are particularly variable [67], which are called complementarity-determining regions (CDRs) with a higher variety of amino acids than the rest parts. For a great number of natural antibodies, antigen-binding sites mainly rely on the CDRs of the heavy chain that some mutations can be introduced into the CDRs of the light chains for dual specificity without weakening the efficiency of antigen binding. Thus, the proof-of-concept study utilized the light chain CDRs of anti-HER-2 antibody Herceptin as a template to select mutations that might bind to a second antigen via phage display technology. After mutations of eleven amino acid residues in light chain CDRs, the antigen-binding sites of Herceptin also bind to VEGF [68]. Overall, the two variant regions of the antibody generated by two-in-one has the same sequence with the ability of dual affinity (dual-acting Fab). In addition, Lee et al. also selected mutations in the CDRs of heavy chains of IL-4 antibody to allow a second binding ability of IL-5 [69]. RG-7597, targeting EGFR and HER3, produced on the two-in-one platform by Genentech, is now undergoing clinical study in phase II for the treatment of head and neck, as well as colorectal cancers (Table 1) [70].

## 3. The Mechanism of BsAbs in Tumor Therapy

### 3.1. Recruiting and Activating Immune Cells

Immune cells play a vital role in the treatment of cancer. Recently, immune checkpoint inhibitors of programmed death-1 (PD-1) and programmed death ligand-1 (PD-L1) have made a breakthrough in the treatment of various solid tumors like malignant melanoma, renal cancer, and NSCLC [71–73]. Immunotherapy represented by chimeric antigen receptor T cell (CAR-T) has also become a new hope for patients with hematological tumors [74–77]. BsAbs have an ability to bind to two different targeting sites, some of which can simultaneously bind to the tumor antigen on the surface of tumor cells as well as another antigen on the surface of immune cells. Mature T cells labeled with CD3 play an important role in the immune response, which have a strong antitumor effect and are widely present in the systemic blood circulation, and become the preferred target for effector cells [78]. It is difficult for immune cells to concentrate on the lesions to work when some cells in the body become cancerous. There are two reasons as follows. First, tumor cells inhibit the activation of T cells. Second, there exist few Fc receptors on the surface of T cells that it is hard to connect tumor cells with natural antibodies [79]. BsAbs can tightly connect tumor cells with T cells by the dual specificities of binding tumor antigens and T cell surface molecules at the same time, so BsAbs can quickly recruit T cells to tumor tissues and eliminate them effectively [80]. Otherwise, BsAbs motivate the function of tumor killing by NK cell recruitment via targeting CD16 or by activating immune cells such as monocytes, macrophages, and dendritic cells [81, 82]. Although the potential for immunogenicity of antibody is an ever-present concern during the development of biopharmaceuticals [83], humoral response to the bispecific antibody catumaxomab could be associated with beneficial humoral effects and prolonged survival of patients with ovarian, nonovarian, or gastric cancers [84]. These interesting results suggested that the immunogenicity of bispecific antibody might be beneficial for the treatment of cancer, and the human anti-mouse antibody- (HAMA-) positive patients might be having a better immune microenvironment than HAMA-negative patients.

### 3.2. Blocking Tumor Dual Signaling Pathway

The occurrence of tumor involves a variety of disease-related signaling pathways, and tumor cells utilize the way of switching signaling pathways to achieve immune escape and prevent damage from drugs. When blocking a single signaling pathway, tumor cells continue to grow by upregulating the expression of other signal molecules in the same or other pathways. Furthermore, the resistance of monospecific antibodies will inevitably take place even if these drugs are demonstrated effective at first. However, BsAbs can achieve a more obvious shrinkage of tumors and delay the drug resistance by targeting dual signals. Some BsAbs reduce growth or immune escape of tumor cells by simultaneously blocking ligands and corresponding receptors of the same signaling pathway [85–87]. For example, PD-L1 protein with overexpressed on tumor cells could bind to the PD-1 on the T cell surface, which could subsequently inactivate T cells, causing the failure of T cells to correctly recognize and clear tumor cell. BsAbs of PD-1/PD-L1 blocking can reactivate T cells to produce more powerful antitumor activities [88]. Other BsAbs target two different antigens of the same tumor cell to increase the specificity and binding affinity of the antibody and subsequently enhance the efficacy of antitumor therapy by simultaneously blocking two signaling pathways which are important for tumor development and metastasis.

## 4. Concluding Remarks

Antibodies have been widely used for clinical applications due to safety and efficacy, which have become the standard drugs for the treatment of many diseases. At the end of 2017, the FDA has approved the applications of 71 antibodies and 8 antibody-like drugs [89, 90]. The global market of antibodies is also expanding from $3 billion in 2000 to $91.63 billion in 2015, a 30-fold increase over 15 years, with an average annual growth rate of 25.6%. Global antibody drug sales of 2017 have already exceeded $100 billion mainly in cancer fields. However, for many solid tumors such as lung cancer, breast cancer, and colorectal cancer, targeting only one antigen is far from enough to prevent tumor progress and drug resistance.

The idea of developing BsAbs emerged half a century ago, and genetic engineering technology makes BsAbs available that there spring up 23 platforms with generation of 62 BsAb molecules. Additionally, more than 50 BsAbs are in the clinical trials and a majority of them are showing good therapeutic effects in preclinic and clinic trials. Bi-/multispecific antibodies are becoming the focus of tumor therapy and may become standard treatment for cancer diseases in the near future. Advances in BsAb engineering have marked a new era of antibodies based on the idea of activating immune system by T cell recruitment in tumor therapy. The newly emerging technologies of BsAb assembly and coexpression *in vitro*, with simplification and high controllability of the process, are easier to achieve accurate assembly of heterologous antibodies. Although there is still a long process for wide use of BsAbs, growing evidences showed that BsAb would be the next generation antibody and a promising reagent against a variety of diseases.

## Figures and Tables

**Table 1:** Immunoglobulin gamma-like bispecific antibody formats.

Format	Company	Publication date		Molecule	Targets	Function	Indication	Clinical trials	Company	Publication number
(A) Immunoglobulin gamma-like asymmetric bispecific antibodies										
Knobs-into-holes (KiH)	Genentech	06 September 1996		(Including in the CrossMAb)						
CrossMAb	Roche	02 July 2009		RG-6026	CD3×CD20	T cell recruitment	Relapsed or refractory non-Hodgkin's lymphoma	I	Roche	WO2016020309 A1
				RG-7221	Angiopoietin 2×VEGF	2-ligand inactivation	Colorectal cancer	II	Roche	WO2011117329 A1
							Solid tumors	I		
				RG-7386	FAP×DR5	Tumor site-specific cell apoptosis	Solid tumors	I	Roche	WO2014161845 A1
				RG-7716	Angiopoietin-2×VEGF	2-ligand inactivation	Diabetic macular edema, wet age-related macular degeneration	II	Roche	US20170260265 A1
				RG-7802	CEA×CD3	T cell recruitment	Solid tumors	I	Roche	WO2013026833 A1
				RG-7828	CD3×CD20	T cell recruitment	Chronic lymphocytic leukemia, non-Hodgkin's lymphoma	I	Genentech	WO2016204966 A1
Triomab quadroma	Fresenius Biotech, TriOn Pharma	14 December 1995	Catumaxomab	EpCAM×CD3	T cell recruitment, Fc-mediated effector function	Malignant ascites, EpCAM-positive gastric and ovarian tumors	Marketed, approved in 2009 by the European Medicines Agency	Neovii Biotech/TriOn Pharma	WO2002020039 A3
			Ertumaxomab	HER2×CD3	T cell recruitment, Fc-mediated effector function	Her2-positive breast cancer	II	Fresenius/TriOn Pharma	US20170210819 A1
			FBTA05	CD3×CD20	T cell recruitment	Lymphoma	I/II	Fresenius/TriOn Pharma	WO20080220568 A1
FcΔAdp	Regeneron	29 December 2010	REGN-1979	CD3×CD20	T cell recruitment	Non-Hodgkin's lymphoma, B cell lymphoma, acute lymphoblastic leukemia, and chronic lymphocytic leukemia	I	Regeneron	WO2014047231 A1
Asymmetric reengineering technology-immunoglobulin (ART-Ig)	Chugai	12 October 2006	Emicizumab	FIXa×FX	2-factor dimerization	Hemophilia A	Marketed, approved in 2017 by the United States Food and Drug Administration	Roche, Chugai (Tokyo)	WO2006109592 A1
			ERY-974	CD3×GPC3	T cell recruitment	Solid tumors	I	Chugai	WO2011078332 A1
BiMAb	OncoMed	24 February 2011	OMP-305B83	DLL4×VEGF	2-ligand inactivation	Solid tumors	I	OncoMed	WO2013044215 A9
Biclonics	Merus	24 October 2013	MCLA-117	CLEC12A×CD3	T cell recruitment	Acute myeloid leukemia	I	Merus	WO2014051433 A1
			MCLA-128	HER2×HER3	2-receptor tyrosine kinase inactivation	Solid tumors	I/II	Merus	WO2015130173 A1
			MCLA-158	Lgr5×EGFR	2-receptor tyrosine kinase inactivation	Solid tumors	I	Merus	WO2016093023 A1
Bispecific Engagement by Antibodies based on the T cell receptor (BEAT)	Glenmark	27 December 2012	GBR-1302	HER2×CD3	T cell recruitment	HER2 positive cancers	I	Glenmark	WO2015063339A1
DuoBody	Genmab	29 December 2011	JNJ-61186372	EGFR×cMET	2-receptor tyrosine kinase inactivation	Non-small-cell lung cancer	I	Janssen, Genmab	WO2014081954 A1
			JNJ-63709178	CD3×CD123	T cell recruitment	Acute myeloid leukemia	I	Janssen, Genmab	WO2016036937 A1
			JNJ-61178104	Undisclosed	Undisclosed	Autoimmune disorders	I	Janssen, Genmab	WO2016052071 A1
Azymetric	Zymeworks	28 June 2012	ZW-25	Two nonoverlapping epitopes of HER2	Receptor tyrosine kinase inactivation	HER2-expressing cancers	I	Zymeworks	WO2015077891 A1
XmAb	Xencor	10 March 2011	XmAb-13676	CD3×CD20	T cell recruitment	B cell malignancies	I	Novartis, Xencor	US20170174781 A1
			XmAb-14045	CD3×CD123	T cell recruitment	Hematological malignancies	I	Novartis, Xencor	WO2016086189 A3

(B) Immunoglobulin gamma-like symmetric bispecific antibodies									
Dual variable domain-immunoglobulin (DVD-Ig)	Abbott	18 August 2006	ABT-122	TNF*α*×IL-17A	2-ligand inactivation	Psoriatic arthritis, rheumatoid arthritis	II	AbbVie (Abbott)	WO2014144280 A3
			ABT-165	DLL4 × VEGF	2-ligand inactivation	Phase I in solid tumors/phase II in colorectal cancer	I/II	AbbVie (Abbott)	WO2014071074 A3
			ABT-981	IL-1*α*×IL-1*β*	2-ligand inactivation	Osteoarthritis	II	AbbVie (Abbott)	WO2008082651 A3
			SAR156597	IL4 + IL13	2-ligand inactivation	Idiopathic pulmonary fibrosis	II	Sanofi	US20170145089 A1
			GSK2434735	IL4 + IL13	2-ligand inactivation	Asthma	I	GlaxoSmithKline	US20170136581 A1
FynomAb	Covagen	23 October 2014	COVA-322	TNF*α*×IL-17A	2-ligand inactivation	Plaque psoriasis	I/II	Covagen	WO2011023685 A1
Two-in-one/dual action Fab (DAF)	Genentech	18 December 2008	RG-7597	EGFR×HER3	2-receptor tyrosine kinase inactivation	Head and neck, colorectal cancers	II	Genentech, Roche	WO2010108127 A1
